# Investigation of the role of southwestern Asia dust events on urban air pollution: a case study of Ahvaz, a highly polluted city

**DOI:** 10.1038/s41598-025-07634-x

**Published:** 2025-07-01

**Authors:** Mehdi Hamidi, Tahoora Ghobadi, Yaping Shao, Bijan Fallah, Masoud Rostami, Rui Mao

**Affiliations:** 1 Faculty of Civil Engineering, Babol Noshirvani University of Technology, Babol, Iran; 2 Institute for Geophysics and Meteorology, University of Cologne, Cologne, Germany; 3 Potsdam Institute for Climate Impact Research (PIK), Potsdam, Germany; 4 German Climate Computing Center (DKRZ), Hamburg, Germany; 5 Laboratoire de Météorologie Dynamique (LMD), Sorbonne University (SU), Ecole Normale Supérieure (ENS), Paris, France; 6 School of National Safety and Emergency Management, Beijing Normal University, Beijing, 100875 China

**Keywords:** Dust events and particle matters, Middle east and southwestern Asia, Ahvaz, Aerosol composition, Hoffmann classification, Environmental sciences, Natural hazards

## Abstract

Investigating aerosol composition and particle dynamics in densely populated and polluted urban centers is crucial for understanding and managing urban air quality. Ahvaz, in southwestern Iran, consistently ranks among the most polluted cities globally, primarily due to high PM_10_ concentrations. This study analyzes trends in suspended particle concentrations in Ahvaz over a 12-year period (2008–2019) to identify the contributions of natural and anthropogenic sources to air pollution. Diurnal, monthly, and annual variations in PM_10_ and PM_2.5_ levels were examined, revealing key insights into the city’s pollution dynamics. Diurnal PM_10_ peaks around noon (232 µg/m^3^), mainly driven by natural dust sources, with minimal anthropogenic impact indicated by similar weekend and weekday concentrations (only 1.5% difference). Monthly analysis reveals significant dust activity in June and July (maximum PM_10_ concentration of 388.18 µg/m^3^), while higher PM_2.5_ levels in winter (average 54.8 µg/m^3^) are attributed to fossil fuel combustion. The PM_2.5_/PM_10_ ratio (mean = 0.24) highlights the dominance of coarse particles from dust events, especially in summer. The Hoffmann classification identifies 3425 dusty days in the study period, with PM_10_ levels notably higher due to dust sources in southern Iraq and southwestern Iran. Seasonal wind patterns, particularly Shamal winds, facilitate dust transport, corroborated by Windrose and PM_10_ rose data. The study underscores the need for regional dust suppression strategies in southern Iraq and southwestern Iran to mitigate air pollution in Ahvaz, highlighting the importance of regional cooperation.

## Introduction

Air pollution has been considered one of the serious environmental problems in recent years, and finding a solution to overcome this problem is an important challenge for the scientific communities. The PM_10_ particle concentration is an important parameter in pollutant analysis and is generally produced by natural resources, i.e. dust events, and anthropogenic factors, i.e. industrial processes, activities of plants, construction, and vehicle transportation^1^. Many strategically important and polluted cities of the world suffer from pollutants originating from natural or anthropogenic sources, and investigating the most effective resources can help decision-makers find a solution to overcome the destructive effects of the pollutants. Ahvaz is one of the most important and populous cities in southwestern Iran and the Middle East and has suffered air pollution during the past decades. Due to its arid and semi-arid climates and its proximity to the Iraq and Kuwait dust sources, it is always exposed to dust events that enter Iran^2,3^. The high frequency of dust plumes entering western and southwestern parts of Iran, along with the arid climate, has caused the city to have significant amounts of airborne particles (PM_10_) throughout the year. According to the World Health Organization (WHO), Ahvaz was identified as the most polluted city in the world in 2011 based on the concentration of PM_10_^4^.

Dust storms, usually containing significant amounts of PM_10_, occur widely around the world and are more common in arid and semi-arid regions^5,6^. The Middle East is one of the main sources of dust in the world due to its arid and desert regions, and dust storms occur in this region all year round^7–9^. Some factors, such as climate changes, successive droughts, and mismanagement of water resources, have resulted in the emission of dust particles from Iraq, Iran, and Kuwait sources in recent years^10,11^. On the other hand, the dust particles originating from Iraq’s dust sources can travel long distances on a regional scale^12^, and dust spread may affect many areas in Iran, especially western and southwestern regions and populated cities like Ahvaz^13^. Hence, dust events can be identified as one of the main hazards in Ahvaz.

Dust events affect human life with its harmful effects in various aspects. Some of the adverse effects of this phenomenon include the shutdown of organizations and industrial units, disruption of power supply systems, reduction of visibility, interruption of transportation, and disruption of the air transportation system. However, the health effects of dust particles have always been considered as the main adverse effect. Studies on the effect of PM_10_ on health have indicated a direct relationship between particle concentration, mortality, and cardiovascular and respiratory diseases^14,15^. It has been reported that 630 people have died on average in Ahvaz each year due to exposure to these particles during 2009 and 2014. The incidence of respiratory and cardiac diseases has been significantly increased at high concentrations^16^. Another hazard of suspended particles is their ability to carry heavy metals. These particles are composed of potentially hazardous elements at high concentrations in many cases. During dust events and increasing concentration of suspended particles, the concentration of heavy metals in the air of Ahvaz increases several times the allowed limit, leading to irreversible effects on the health of residents and the environment^17^. The mentioned reasons could indicate the necessity of investigating Ahvaz air quality under the dust particles’ effects and the relationship between PM_10_ and dust activities.

Many studies have been conducted on dust’s chemical, physical, biological, and bacterial properties in Ahvaz^2,18–20^. The literature review on suspended particles in Ahvaz shows that this phenomenon has been studied more in the fields of synoptic, environments, and mineralogy. Although the air of Ahvaz often has significant concentrations of suspended particles, few studies have been conducted on trends of annual, seasonal, and monthly changes in these particles. Most of the available studies are case studies and have addressed dust in Ahvaz in a short period, and few studies have investigated the trend of changes in the long period^16,20^. Also, the existing studies have focused only on very high concentrations of suspended particles, and lower concentrations have been ignored. Since the mean daily concentration of suspended particles in Ahvaz on almost all days of the year is above the allowed standards and significantly affects air quality, it is necessary to investigate the trend of changes in particle concentration over a long period^16,21,22^.

This study employs a novel methodology to investigate the key parameters influencing air pollution in Ahvaz, a strategically important, densely populated, and polluted city. Beyond addressing the necessity of studying this strategic Middle Eastern city to improve its air quality, this research provides a framework for examining the roles of anthropogenic and natural sources in urban pollution globally. Given the significance of pollution in Ahvaz, characterized by high PM_10_ concentrations, this study analyzes the trends in daily, monthly, and annual PM_10_ concentration changes over a 12-year period (2008‒2019). The investigation also examines the relationship between particle concentrations and dust events by comparing changes on dusty and normal days. Additionally, the study explores the impact of meteorological conditions on air pollutants by analyzing the relationship between suspended particles and parameters such as wind speed and direction. To further elucidate the correlation between PM_10_ concentrations in Ahvaz and regional dust sources, a correlation analysis between Ahvaz’s PM_10_ data and dust activity in southern Iraq was conducted.

## Materials and methods

### Study area

**Fig. 1:**
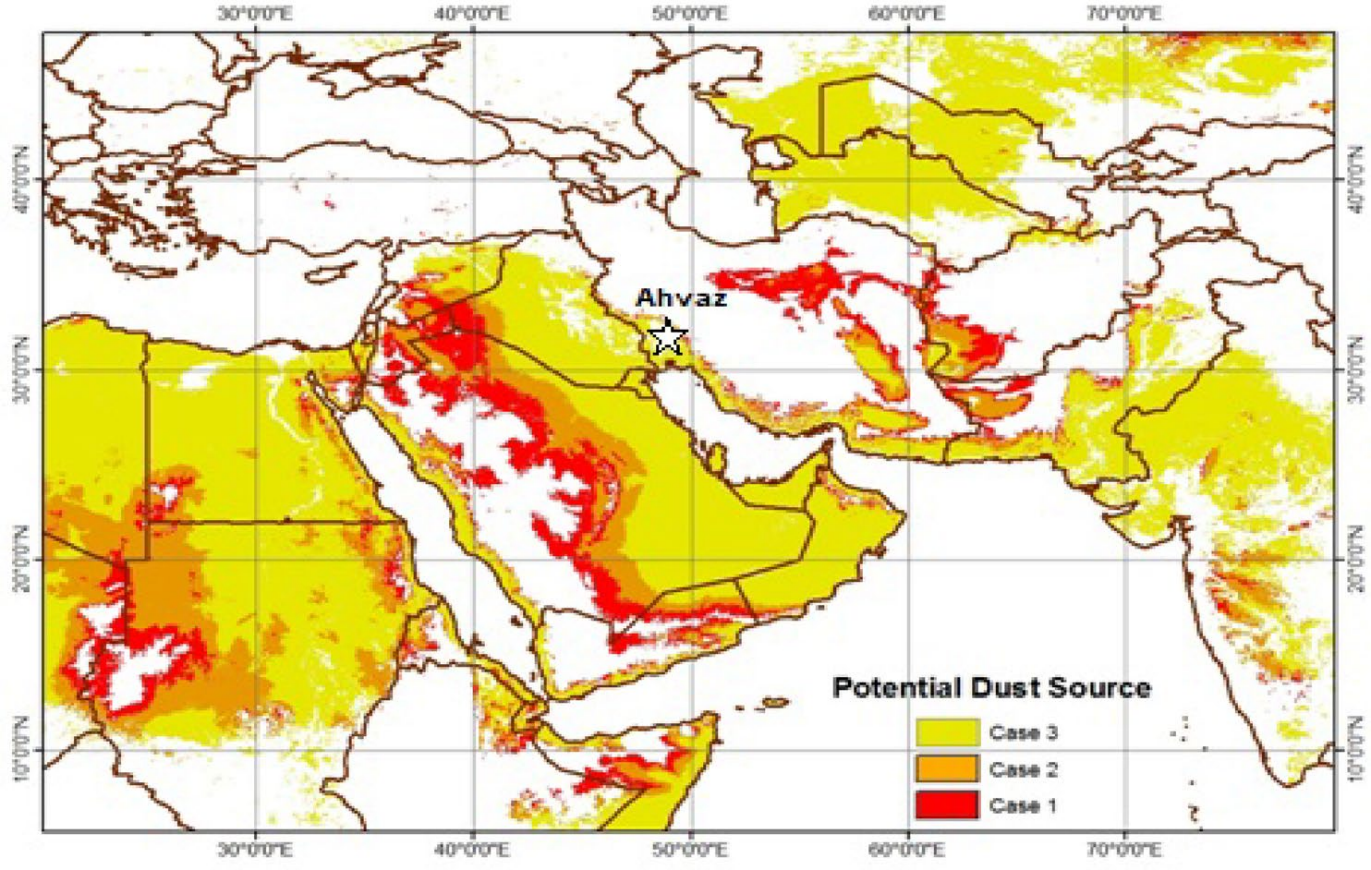
Geographic location of Ahvaz and its stations relative to potential dust source regions in the Middle East and Western Asia. The shaded areas represent the dust source regions as identified in Cases 1, 2, and 3. For Case 2 (LAIc = 0.3, Hc = 700), the expanded region compared to Case 3 (LAIc = 0.3, Hc = 500) is highlighted in orange. For Case 1 (LAIc = 0.3, Hc = 900), the expanded region compared to Case 2 is highlighted in red (adapted from Hamidi et al.^23^).

Ahvaz is situated at 31° 50′ N latitude and 49° 11′ E longitude, within the Khuzestan Province plain in Iran. With a population of 1.4 million, it is one of the most densely populated cities among the countries in the northeastern region of the Persian Gulf. Figure 1 shows the location of Ahvaz and its place among the potential dust sources of the Middle East and Western Asia. According to De Martonne’s climate classification, which is based on mean rainfall and temperature, Ahvaz has an arid climate. The city is located near the borders of Iraq, Kuwait, and Saudi Arabia, the main sources of dust particles in the Middle East and Western Asia. Ahvaz is significantly affected by dust intrusions from these countries through Iran’s western and southwestern borders^23^. Additionally, as illustrated in Fig. 1, the surrounding area of Ahvaz comprises deserts prone to wind erosion, which can act as dust sources.

### Methodology

The data utilized in this study comprised air pollution measurements from monitoring stations in Ahvaz and meteorological data, including prevailing wind speed and direction. Particulate matter concentrations were recorded at four stations over 12 years by the Environmental Protection Organization of Ahvaz. Particle concentrations were measured using the Beta Attenuation Method (Metone BAM-1020 unit), which automatically records concentrations in micrograms per cubic meter under local or standard temperature and pressure conditions. The device operates by emitting beta waves from a carbon source through a specified air volume containing dust particles, measuring the density of particles condensed on the filter. It records average hourly particle concentrations. This method is widely used globally for measuring suspended particle concentrations and is considered one of the most reliable air monitoring tools. The BAM-1020 unit has been employed in numerous studies to monitor particulate matter concentrations^24^. These PM_10_ and PM_2.5_ data were obtained from the mentioned organization and the mean values of diurnal, monthly, and annual variations were used for detailed analysis.

Data from the Meteorological Organization of Ahvaz were utilized to provide necessary meteorological information for the study period, including horizontal visibility, wind speed, and wind direction. These data were collected at 3-h intervals at an altitude of 10 m above ground level. Information on prevailing winds, the wind rose of Ahvaz, and variations in wind speed during the study period were obtained. Wind rose diagrams were generated using WRPLOT software, which is used for statistical evaluation of wind speed and direction. Wind data recorded during dust events were used to generate PM_10_ rose diagrams, which help identify the wind directions that significantly influence the entry and transport of dust in the region. Finally, a comparative analysis was conducted between pollutant concentrations on dusty and normal days, along with the size ratios of particulate matter (PM) to determine the contribution of dust particles to overall particle concentrations. Studies have shown that suspended particles from natural sources are generally coarser than those from anthropogenic activities. Particles with diameters between 2.5 and 10 µm are primarily attributed to mineral dust, whereas particles smaller than 2.5 µm are primarily associated with anthropogenic sources^1,25^. Therefore, the ratio between concentrations of suspended particles of different sizes can serve as a parameter to distinguish their sources. According to this criterion, low PM_2.5_/PM_10_ ratios suggest a greater contribution of natural sources compared to anthropogenic sources. Furthermore, Hoffmann’s classification system was employed to categorize dust events and examine their association with PM_10_ concentration. In addition, to evaluate the role of southern Iraq dust sources on Ahvaz air quality, a correlation analysis between Ahvaz’s PM_10_ data and dust activity in the mentioned area was conducted.

## Results and discussion

### Diurnal variation in PM_10_ concentration

**Fig. 2:**
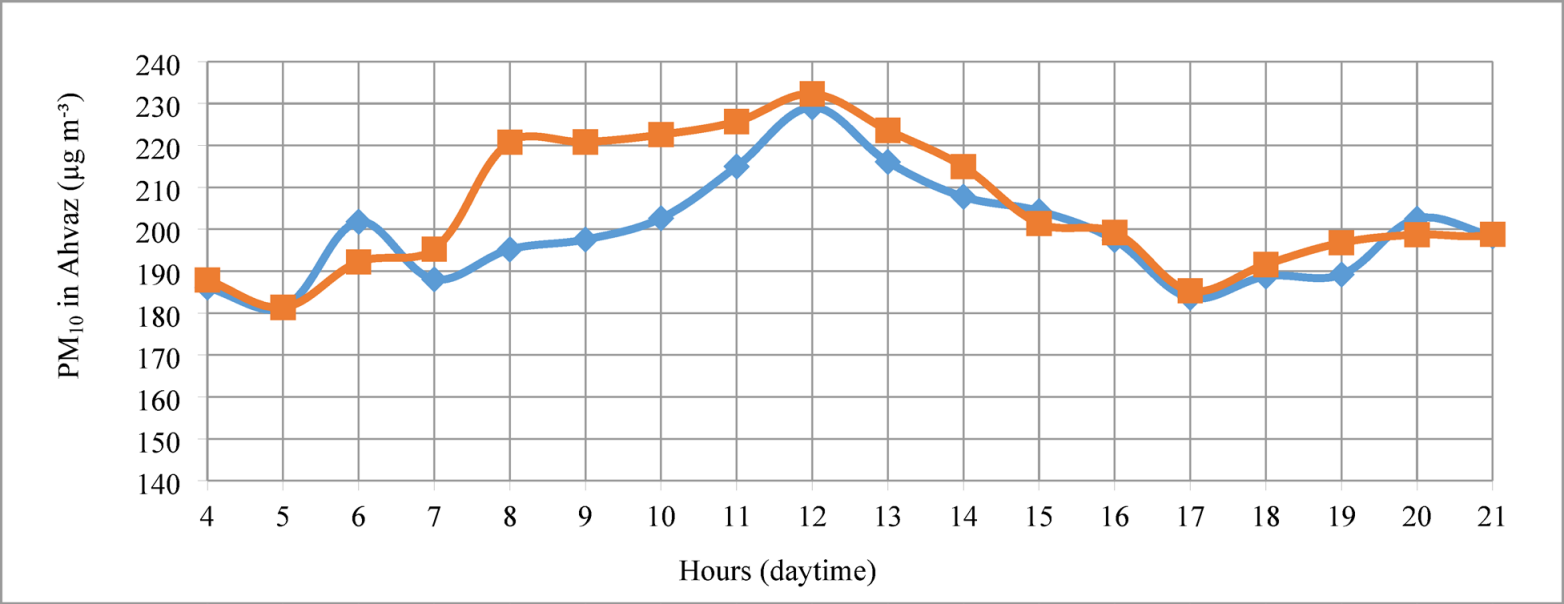
Diurnal changes in PM_10_ concentration over the study period in Ahvaz (µg m^−3^). Data are shown for all weekdays (red colored) and weekends (blue colored).

To assess pollution levels and their relationship with nearby dust sources, diurnal variations in PM_10_ concentrations in Ahvaz were analyzed over a 12-year period. Figure 2 illustrates the hourly trends in daytime PM_10_ concentrations for weekdays and weekends. In fact, It represents the average PM_10_ concentration for a specific hour, calculated over the entire 12-year study period. The data indicate that peak PM_10_ concentrations occur near noon local time (232 µg m^−3^). The particle concentration shows a relatively uniform increase in the morning, reaching its maximum at noon, followed by a uniform decline later in the day. The concentration of suspended particles in Ahvaz can be influenced by daily activities, meteorological conditions, and wind speed. Analysis of diurnal wind speed trends during the study period revealed a relationship between surface wind speed and PM_10_ concentrations. According to Ahvaz meteorological data, wind speed increases during the morning hours, peaking at noon, which aligns with the highest PM_10_ concentrations. The correlation between PM_10_ concentrations and natural sources, which has been documented in previous studies^26,27^ and in this study, can be attributed to dust emissions from surrounding dust sources.

The contribution of dust events and anthropogenic factors varies based on the population density, industrial characteristics of the study area, and the frequency of dust events. In large, densely populated cities with significant vehicle traffic, the difference in PM_10_ concentrations is more pronounced between weekdays and weekends^28^. However, the diurnal variation of PM_10_ in Ahvaz (Fig. 2) shows that the peak PM_10_ concentrations on weekends and weekdays differ by only 1.5%, indicating no significant relationship between peak PM_10_ levels and anthropogenic emissions such as vehicle traffic or industrial activities. Conversely, the average PM_10_ concentration during the early hours of the day (6‒12 a.m.) is 204 µg m^−3^ on weekends, which is 5% lower than the 215 µg m^−3^ observed during the same period on weekdays. This suggests that while anthropogenic sources may slightly influence pollutant concentrations on working days, they do not significantly affect dust pollution in Ahvaz. Instead, natural sources play a more critical role in the city’s air pollution.

### Monthly PM concentrations and PM_2.5_/PM_10_ ratio

Analyzing the PM concentration and the PM_2.5_/PM_10_ ratio is a suitable way to determine the natural or anthropogenic sources of pollutants. Table 1 presents a statistics summary of PM_10_ and PM_2.5_ concentrations over the study period. The mean concentrations of PM_10_ and PM_2.5_ during the sampling period were 223.4 µg m^−3^ and 52.3 µg m^−3^, respectively. The maximum hourly concentrations for PM_10_ and PM_2.5_ were 10,000 µg m^−3^ and 940.27 µg m^−3^, respectively, both occurring in June. The average standard deviations for PM_10_ and PM_2.5_ concentrations were 387 and 82 µg m^−3^, respectively. These results are corroborated by previous studies^20,21^, but this study encompasses a broader data range, potentially increasing its reliability.

**Table 1:** Statistics summary of PM_10_ & PM_2.5_ concentrations in Ahvaz during the study period (μg m^−3^).

Month	PM_10_					PM_2.5_				
	Mean	Min	Max	Median	SD	Mean	Min	Max	Median	SD
January	177.09	9.3	8056	100.00	503.86	65.69	7.6	317.24	26.30	112.92
February	265.19	11.6	10,000	154.23	752.64	70.35	4.2	672.01	38.01	134.46
March	179.08	19.2	8200	105.00	469.24	47.2	5.3	344.00	26.25	106.72
April	192.43	17.6	9334	112.45	348.28	36.43	4.2	340.02	28.34	67.57
May	293.92	25.9	9921	139.96	351.99	45.32	4.6	868.96	38.76	63.93
June	346.90	28.6	10,000	210.90	412.58	64.63	8.1	940.27	51.06	78.26
July	388.18	23.3	10,000	202.37	502.5	72.15	9.6	547.23	49.43	101.54
August	189.59	21.1	8440	144.00	320.63	43.61	9.4	186.00	41.30	66.72
September	188.04	6.1	7350	136.00	294.74	37.61	7.6	213.00	26.10	58.67
October	167.46	4.3	6874	132.27	209.07	41.98	4.4	318.32	33.20	50.38
November	143.77	3.7	6381	101.33	229.38	46.6	3.2	322.08	28.34	65.24
December	148.97	6.6	6915	101.56	255.27	57.07	4.3	385.60	21.67	77.52

**Fig. 3:**
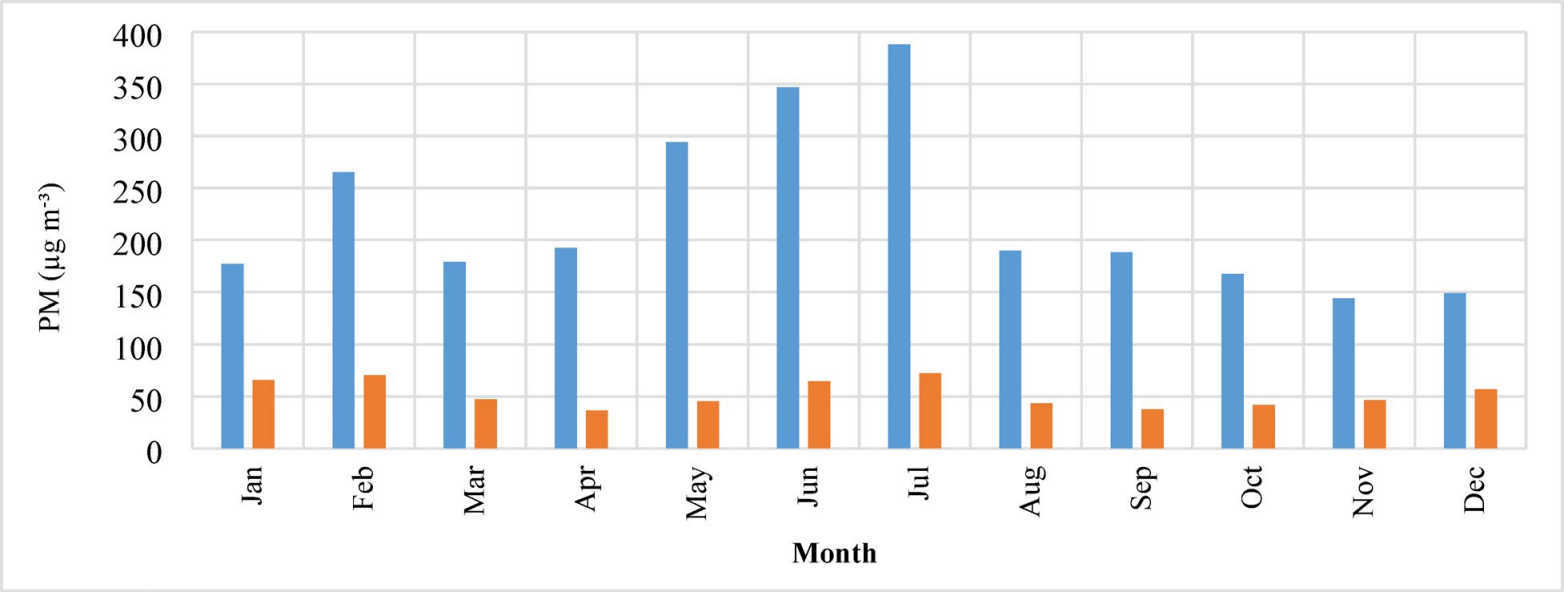
Monthly variations of PM_10_ (blue) and PM_2.5_ (red) Concentration over the study period in Ahvaz.

The trend of changes in the mean monthly concentrations of PM_10_ and PM_2.5_ during the study period is illustrated in Fig. 3. The data reveal that the highest PM_10_ concentration occurred in July (388.18 µg m^−3^), while the lowest concentration was observed in November (143.7 µg m^−3^), and PM_10_ concentration in March has an increasing trend, with a peak in June and July. Additionally, the pattern of monthly variations in particle concentrations across different years is consistent, with the highest concentrations typically recorded in late spring and early summer.

An investigation of PM_2.5_ concentrations revealed that the mean concentration of particles during the cold season (in this area, October to March) was 54.8 µg m^−3^, higher than the warm season’s (in this area, April to September) mean concentration of 49.95 µg m^−3^. This increase is likely due to the higher combustion of fossil fuels for heating during the cold season. Similar seasonal trends in PM_2.5_ concentrations have been observed in other studies^29^. Conversely, an analysis of PM_10_ concentrations showed that the mean concentration during the warm season (April to September) was 266.5 µg m^−3^, compared to 180. µg m^−3^ during the cold season (October to March). This difference is likely due to increased dust activity in the Middle East and Western Asia during the warm season, which significantly contributes to PM_10_ levels in Ahvaz^30,31^.

**Fig. 4:**
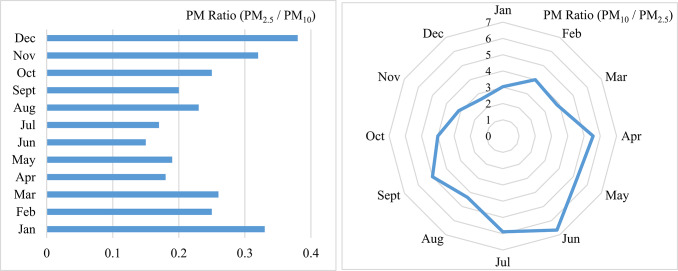
Monthly PM_2.5_/PM_10_ (left) and PM_10_/PM_2.5_ (right) ratio during the study period in Ahvaz.

The PM_2.5_/PM_10_ ratio is a valuable tool for identifying the sources of particulate matter. Elevated ratios typically indicate contributions from combustion sources such as vehicle emissions, industrial processes, and residential heating, which predominantly emit finer particles (PM_2.5_). Conversely, lower ratios suggest contributions from natural sources such as dust storms and soil erosion, which generate coarser particles (PM_10_). This ratio provides critical information for developing air pollution control strategies. A high PM_2.5_/PM_10_ ratio would suggest focusing policies on reducing emissions from combustion sources, while a low ratio requires efforts toward controlling dust emissions. The analysis of the PM_2.5_/PM_10_ ratio based on monthly mean concentrations is presented in Fig. 4. The mean PM_2.5_/PM_10_ ratio throughout the study period was 0.24, with monthly variations ranging from 0.15 to 0.38. The highest mean ratio (0.38) occurred in December, while the lowest ratios were observed in June and July with values 0.15 and 0.17, respectively. The lower ratios during June and July can be attributed to increased dust activity, indicating a higher presence of coarse particles. This suggests that Ahvaz’s suspended particles are predominantly coarse, consistent with regions affected by natural dust events. In contrast, areas with pollution primarily from anthropogenic emissions report higher PM_2.5_/PM_10_ ratios, such as 0.71, as observed by Cheng et al.^32^, highlighting the significant contribution of dust events to Ahvaz’s air pollution. To further elucidate the contribution of natural dust sources to air pollution in Ahvaz, a PM_10_/PM_2.5_ ratio diagram is also included in Fig. 4. This diagram illustrates that in certain months, such as June and July with higher dust activity in southwestern Asia, this ratio approaches approximately 6.5, which can visually reflect the high contribution of natural pollutant sources compared to the anthropogenic.

### PM_10_ concentration and dust in Ahvaz

To investigate the relationship between PM_10_ concentrations in Ahvaz and regional dust activities, an additional analysis was conducted for the study period. The number of dusty days and corresponding particle concentrations were determined using the Hoffmann classification method, as detailed in Table 2. This classification method is based on parameters such as visibility, suspended particles, and wind speed, enabling the categorization of dust events according to their intensity^33^. It classifies dust events into five distinct categories: dusty air (DA), light dust storms (DS1), dust storms (DS2), strong dust storms (DS3), and severe dust storms (DS4). The analysis revealed 3,425 dusty days, accounting for approximately 78% of the study period. This extensive occurrence of dusty days underscores the significant influence of dust events on PM_10_ levels in Ahvaz.

**Table 2:** Number of dusty days based on classification of Hoffmann.

Category	Number of Dusty days	percentage of dust days respect to study period
Dusty air (DA)	2140	48.9
Light dust storm (DS1)	973	22.2
Dust storm (DS2)	287	6.5
Strong dust storm (DS3)	21	–
Serious dust storm (DS4)	4	–

For further analysis of dusty days, PM_10_ concentrations were assessed on both normal and dusty days using the Hoffmann classification, categorized into cold seasons (autumn and winter), and warm seasons (spring and summer). Table 3 presents these findings. PM_10_ concentrations for normal days were 136 µg m^−3^ in warm seasons and 113 µg m^−3^ in cold seasons, while on dusty days, concentrations were 798 µg m^−3^ and 647 µg m^−3^, respectively. The substantial increase in PM_10_ levels, particularly in warm seasons, is attributed to frequent dust events in southern Iraq and southwestern Iran, resulting in a six-fold rise in pollutant concentrations compared to normal days.

**Table 3:** PM_10_ concentration in Ahvaz during normal and dusty days based on cold and warm seasons (µg m^-3^).

Seasons	Weather condition	Mean	Min	Max	SD
Warm seasons	Normal	136	6	184	57
	Dusty	798	236	10,000	832
Cold seasons	Normal	113	4	176	49
	Dusty	647	218	10,000	586

This trend of increased dust activity during warm periods in the Middle East and southwestern Asia has been documented by prior research. Middleton reported that spring and summer are the seasons with the highest frequency of dust storms in Mesopotamia and Iraq^34^. Additional studies have observed maximum PM_10_ concentrations in neighboring countries such as Saudi Arabia, Kuwait, and Iraq during the summer, correlating with heightened dust events in the Middle East^35^. Shao indicated that dust activity in the region increases in spring and reaches its peak in June and July^36^. The mentioned patterns are consistent with the findings of this study and a corresponding increase in PM_10_ concentrations. This consistency is shown in Fig. 2 and indicates the key role of southern Iraq and southwestern Iran dust events in PM_10_ particle concentration in Ahvaz. In a similar study conducted on Ahvaz, the highest concentration was observed in July, and the pollutant source was from dust sources in Iraq^21^. Adverse climatic conditions in Western Asia and the Middle East significantly contribute to high concentrations of suspended particles in Ahvaz. Seasonal wind patterns, particularly in warmer months, exacerbate this issue. The Shamal wind, prevalent from June to September with peaks in June and July, transports large amounts of dust to southwestern Iran, including Ahvaz, thereby increasing particle concentrations^23,37–39^. Similar increases in particle concentrations during warmer months are observed in neighboring countries such as Saudi Arabia, Iraq, and Kuwait^40^. Moreover, rising temperature and wind speed trends from 2000 to 2017 in the Middle East suggest an escalation in dust activity^40^. Consequently, it could be said that future increases in PM_10_ concentrations in Ahvaz appear inevitable given current wind directions and dust transport paths.

**Fig. 5:**
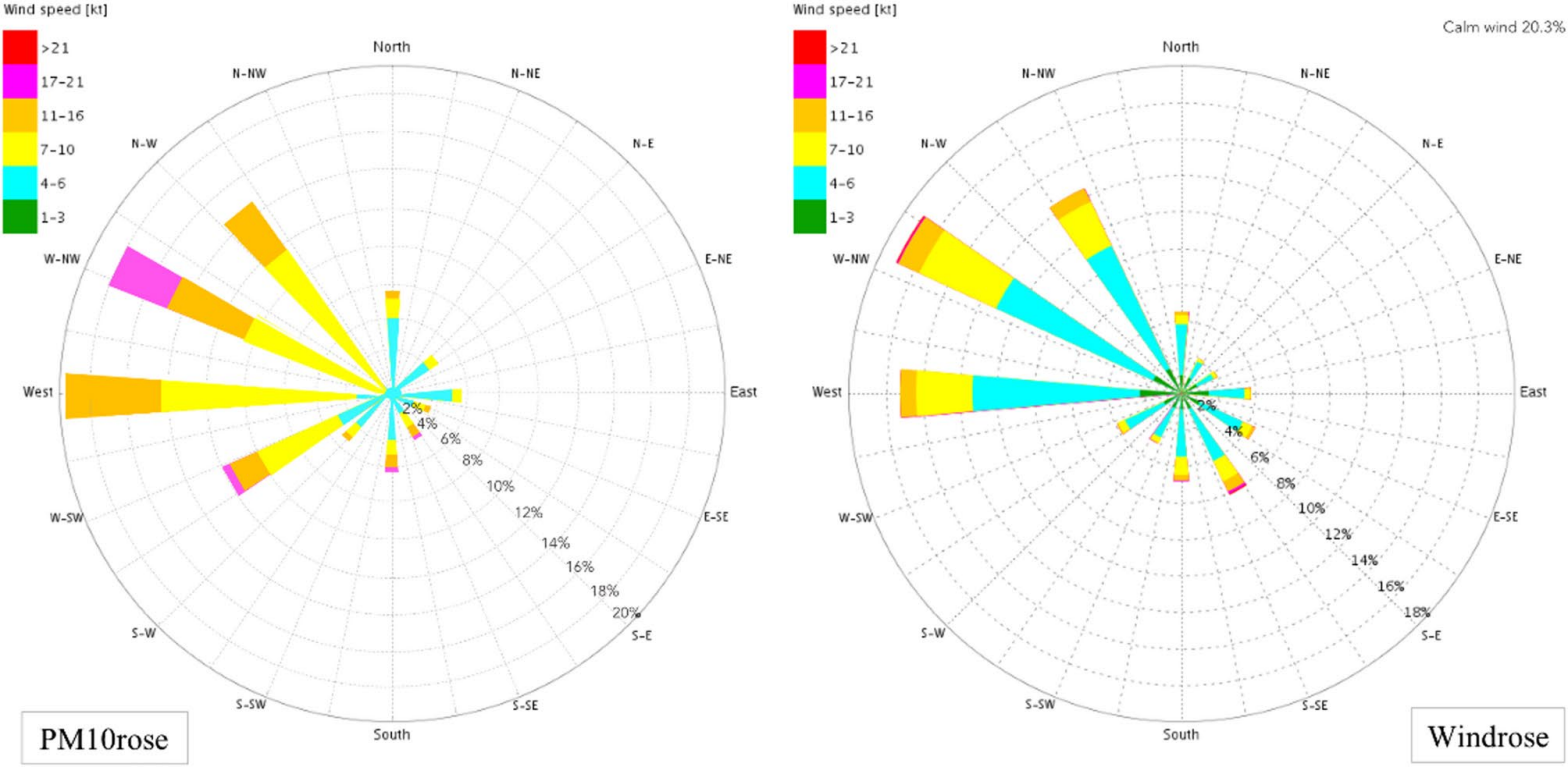
Wind rose (right) and PM_10_ rose (left) over the study period in Ahvaz. Wind data on all days and dusty days are used to plot Wind rose and PM_10_ rose, respectively.

For a complementary analysis of dust particle transport pathways and dominant dust sources, the magnitude and direction of wind throughout the study period specifically on dusty days were examined. The results are illustrated in Wind rose and PM_10_ rose diagrams. Wind rose plots are typically used to quickly assess wind patterns in a region, showing wind speed, direction, and frequency using a central coordinate system. Figure 5 presents the Wind rose and PM_10_ rose diagrams for Ahvaz during the study period, with the PM_10_ rose derived from wind data on dusty days. The analysis indicates that the prevailing wind of Ahvaz Meteorological Station originates from the west and northwest, constituting 32% of total winds. Additionally, the PM_10_ rose analysis shows that approximately 69% of winds on dusty days have westerly, northwesterly, and southwesterly directions. This alignment between the annual Wind rose and PM_10_ rose data suggests a consistency in prevailing wind directions contributing to dust transport in the region. Due to Ahvaz’s geographical proximity to northwestern, western, and southwestern neighboring countries, it can be concluded that the prevailing wind directions during dust events transport substantial amounts of mineral dust particles from southern and southeastern Iraq, as well as occasionally from Kuwait and Saudi Arabia, into southwestern Iran and Ahvaz. This dust influx significantly impacts Ahvaz’s atmospheric conditions, resulting in elevated PM_10_ concentrations and detrimental effects on air quality in the city.

### Annual changes in PM_10_ concentration

**Fig. 6:**
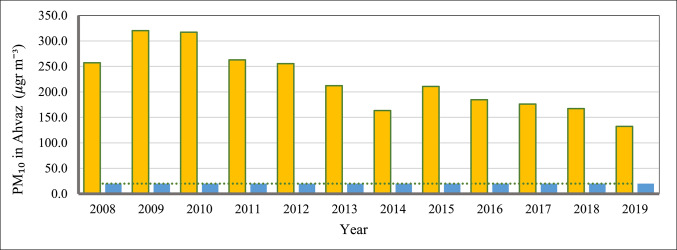
Annual PM_10_ concentration in Ahvaz during 2008–2019. The blue color and horizontal line show the limits for annual average PM_10_ concentration (20 μg m^−3^, following the 2005 Air Quality WHO guideline).

The mean annual PM_10_ concentrations in Ahvaz were analyzed to assess pollution levels and their relationship with surrounding dust sources. Figure 6 illustrates the annual PM_10_ concentrations in Ahvaz from 2008 to 2019. The highest concentration was recorded in 2009 at 320.2 µg m^−3^, while the lowest was 132.4 µg m^−3^ in 2019. The average annual PM_10_ concentration was 222 µg m^−3^. This value is approximately 14.8 times higher than the World Health Organization (WHO) recommended limit of 15 µg/m^3^, indicating severe pollution levels^22^. Similarly, the PM_10_ concentration in Ahvaz exceeds the National Ambient Air Quality Standards (NAAQS) set by the US EPA, and European Environment Agency (EEA) Air Quality Standards, highlighting Ahvaz as one of the most polluted cities globally^41^.

**Fig. 7:**
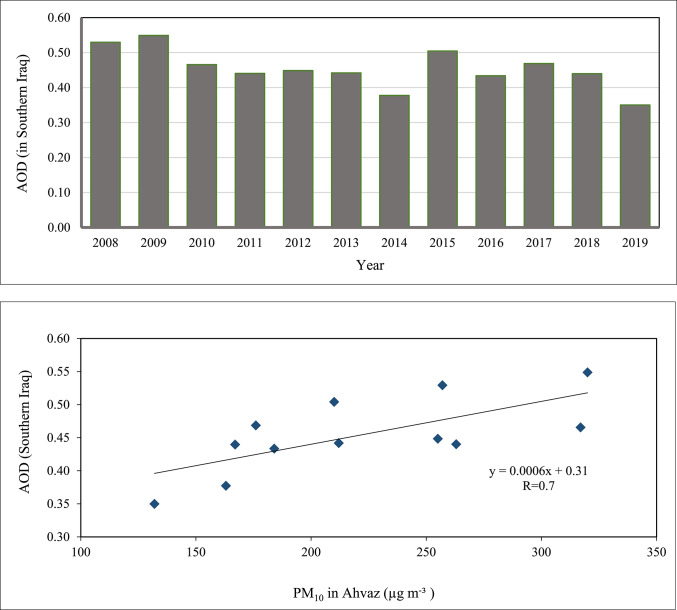
Annual variation of AOD in Southern Iraq and its relationship with Annual PM10 concentration in Ahvaz, Top: Annual variation of AOD in Southern Iraq during 2008‒2019 (adopted from Hamidi and Roshani^31^), Bottom: Correlation analysis Between PM_10_ in Ahvaz and Southern Iraq dust activity.

To analyze the influencing factors on PM_10_ concentrations and the role of natural sources influence in Ahvaz air pollution, the relationship between annual PM_10_ levels in Ahvaz and dust activity in southern Iraq and southwestern Iran during the study period was examined. Studies have shown a high frequency of dust storms in Southern Iraq and Southwestern Iran 2007‒2010^10^ and a reduction between 2011‒2014 and 2016‒2019, which have a similar trend to the annual PM_10_ concentrations in Ahvaz. To evaluate the role of the Southern Iraq dust activity on Ahvaz PM_10_ concentrations, the annual Aerosol Optical Depth (AOD) variation during 2008–2019 was obtained from Hamidi and Roshani^31^ studies. Figure 7 shows the annual variation of dust activity (AOD) in Southern Iraq (top diagram) and the correlation between the dust activities in this area with Ahvaz PM_10_ concentrations (bottom diagram).

The analysis result shows a sufficient correlation between the mentioned parameters and confirms the high influence of dust activity in southern Iraq on PM_10_ concentrations in Ahvaz. Based on the study by Hamidi and Roshani^31^, AOD levels are projected to increase by 10.5% under SSP2-4.5 and 15.2% under SSP5-8.5 scenarios over the next two decades compared to the 2000–2020 baseline. This anticipated rise highlights the potential intensification of dust activity in the region, including Ahvaz, and reinforces the long-term concern illustrated in Fig. 7. In other words, this correlation demonstrates the necessity of finding suitable ways to suppress dust emissions in southern Iraq and surrounding area dust sources to reduce the pollutant concentration in Ahvaz and other polluted cities in the area.

## Conclusion

This study analyzed the variation trends in suspended particle concentrations in Ahvaz, a key city in southwestern Iran, over a 12-year period (2008‒2019) to understand the roles of natural and anthropogenic sources in air pollution. Utilizing data from the Ahvaz Environmental Protection Organization, the research examined diurnal, monthly, and annual variations in PM_10_ and PM_2.5_ levels, revealing significant insights into the city’s pollution situation and sources.

Diurnal PM_10_ variations in Ahvaz showed peak levels around noon, corresponding with increased wind speeds and indicating a major influence from natural dust sources. The negligible difference in PM_10_ peaks between weekends and weekdays (1.5%) and slightly lower early morning concentrations on weekends suggested minimal impact from anthropogenic activities like traffic and industry, underscoring the predominant role of natural dust sources. Monthly analysis of PM_2.5_ and PM_10_ concentrations, and their ratios, identified the high influence of dust activity in June and July in Ahvaz air pollution. Higher PM_2.5_ levels in the cold season were attributed to fossil fuel combustion for heating, while elevated PM_10_ levels in the warm season were linked to dust storms. The PM_2.5_/PM_10_ ratio emphasized the dominance of coarse particles from natural dust events, particularly in the summer. The Hoffmann classification identified 3,425 dusty days, highlighting the significant influence of dust events on Ahvaz’s air quality. PM_10_ levels were notably higher on dusty days, especially in the warm seasons, due to dust sources in southern Iraq and southwestern Iran. Seasonal wind patterns, particularly Shamal winds, exacerbated dust transport to Ahvaz, corroborated by Wind rose and PM_10_ rose data. This analysis indicated westerly, northwesterly, and southwesterly winds are the primary mechanisms of dust transport to Ahvaz. Annual PM_10_ concentrations from 2008 to 2019 revealed severe pollution levels, significantly exceeding WHO and US EPA standards, with a peak in 2009 (320.2 µg m^−3^) and a low in 2019 (132.4 µg m^−3^), averaging 222 µg m^−3^. A good correlation between PM_10_ levels in Ahvaz and dust activity in southern Iraq and southwestern Iran underscored the critical impact of regional dust storms on Ahvaz air pollution. The study concludes that effective dust suppression strategies in southern Iraq and southwestern Iran are essential to reducing air pollution in Ahvaz and similar cities. This necessitates targeted regional cooperation and interventions to mitigate dust emissions and improve air quality. Regional collaboration is crucial for addressing high mineral dust pollution levels, demonstrating that reducing air pollutant concentrations requires a coordinated approach.

## Data Availability

The datasets used and/or analysed during the current study available from the corresponding author on reasonable request.

## References

[1] Querol, X. et al. Speciation and origin of PM10 and PM2.5 in selected European cities. *Atmos. Environ.* **38**(38), 6547–6555 (2004).

[2] Naimabadi, A. et al. Chemical composition of PM10 and it’s in vitro toxicological impacts on lung cells during the Middle Eastern Dust (MED) storms in Ahvaz, Iran. *Environ. Pollut.* **211**, 316–324 (2016). 26774778 10.1016/j.envpol.2016.01.006

[3] Ledari, D. G., Hamidi, M. & Shao, Y. Numerical simulation of the 18 February 2017 frontal dust storm over the southwest of Iran using WRF-Chem, satellite imagery, and PM10 concentrations. *J. Arid Environ.* **196**, 104637 (2022).

[4] Goudie, A. S. Desert dust and human health disorders. *Environ. Int.* **63**, 101–113 (2014). 24275707 10.1016/j.envint.2013.10.011

[5] Wang, S., Wang, J., Zhou, Z. & Shang, K. Regional characteristics of three kinds of dust storm events in China. *Atmos. Environ.* **39**(3), 509–520 (2005).

[6] Huang, J., Wang, T., Wang, W., Li, Z. & Yan, H. Climate effects of dust aerosols over East Asian arid and semiarid regions. *J. Geophys. Res.* **119**(19), 11398–11416 (2014).

[7] Hamidi, M. Atmospheric Investigation of frontal dust storms in Southwest Asia. *Asia-Pac. J. Atmos. Sci.* **55**(2), 177–193 (2019).

[8] Tanaka, T. Y. & Chiba, M. A numerical study of the contributions of dust source regions to the global dust budget. *Global Planet. Changes* **52**(1), 88–104 (2006).

[9] Ginoux, P., Prospero, J. M., Torres, O. & Chin, M. Long-term simulation of global dust distribution with the GOCART model: Correlation with North Atlantic Oscillation. *Environ. Model. Softw.* **19**, 113–128 (2004).

[10] Hamidi, M. The key role of water resources management in the Middle East dust events. *CATENA* **187**, 104337 (2020).

[11] Mohseni, F. & Hamidi, M. Investigating dust storm dynamics: Quantifying terrestrial impacts using WRF-Chem in Arid regions. *Earth**Syst*. *Environ*. 1–16 (2024).

[12] Middleton, N. J. Desert dust hazards: A global review. *Aeol. Res.* **24**, 53–63 (2017).

[13] Ledari, D. G., Hamidi, M. & Shao, Y. Evaluation of the 13 April 2011 frontal dust storm in West Asia. *Aeol. Res.* **44**, 100592 (2020).

[14] Perez, L. et al. Coarse particles from Saharan dust and daily mortality. *Epidemiology* **19**(6), 800–807 (2008). 18938653 10.1097/ede.0b013e31818131cf

[15] Chan, C. C. et al. Increasing cardiopulmonary emergency visits by long-range transported Asian dust storms in Taiwan. *Environ. Res.* **106**, 393–400 (2008). 17959168 10.1016/j.envres.2007.09.006

[16] Maleki, H., Sorooshian, A., Goudarzi, G., Nikfal, A. H. & Baneshi, M. M. Temporal profile of PM10 and associated health effects in one of the most polluted cities of the world (Ahvaz, Iran) between 2009 and 2014. *Aeol. Res.* **22**, 135–140 (2016). 10.1016/j.aeolia.2016.08.006 PMC5422000 28491152

[17] Zarasvandi, A., Carranza, E. J. M., Moore, F. & Rastmanesh, F. Spatio-temporal occurrences and mineralogical-geochemical characteristics of airborne dust in Khuzestan Province (Southwestern Iran). *J. Geochem. Explor.* **111**(3), 138–151 (2011).

[18] Marzouni, M. B. et al. Health benefits of PM10 reduction in Iran. *Int. J. Biometeorol.* **61**(8), 1389–1401 (2017). 28382377 10.1007/s00484-017-1316-2

[19] Heidari-Farsani, M. et al. The evaluation of heavy metals concentration related to PM10 in ambient air of Ahvaz city, Iran. *J. Adv. Environ. Health Res.* **1**(2), 120–128 (2014).

[20] Goudarzi, G. et al. Particulate matter and bacteria characteristics of the Middle East Dust (MED) storms over Ahvaz, Iran. *Aerobiologia* **30**, 345–356 (2014).

[21] Shahsavani, A. et al. The evaluation of PM10, PM2.5, and PM1 concentrations during the Middle Eastern Dust (MED) events in Ahvaz, Iran, from April through September 2010. *J. Arid Environ.* **77**, 72–83 (2012).

[22] World Health Organization. WHO Global Air Quality Guidelines 2021. World Health Organization (2021).

[23] Hamidi, M., Kavianpour, M. & Shao, Y. Synoptic analysis of dust storms in the Middle East. *Asia-Pac. J. Atmos. Sci.* **49**(3), 279–286 (2013).

[24] Hauck, H. et al. On the equivalence of gravimetric PM data with TEOM and beta-attenuation measurements. *J. Aerosol Sci.* **35**, 1135–1149 (2004).

[25] Li, Z. et al. Sources of fine particle composition in New York City. *Atmos. Environ.* **38**(38), 6521–6529 (2004).

[26] Saghafi, M. A. & Aliakbari, B. Investigate diurnal and seasonal variation of wind, the temperature in the surface atmospheric layers in Tehran city. *J. Spatial Anal. Environ. Hazard.* **1**, 17–34 (2014) (**In Persian**).

[27] Wang, P. et al. Impact of meteorological parameters and gaseous pollutants on PM2.5 and PM10 mass concentrations during 2010 in Xi’an. China. *Aerosol Air Qual. Res.* **15**, 1844–1854 (2015).

[28] Bathmanabhan, S. & Saragur Madanayak, S. N. Analysis and interpretation of particulate matter—PM10, PM2.5 and PM1 emissions from the heterogeneous traffic near an urban roadway. *Atmos. Pollut. Res.* **1**(3), 184–194 (2010).

[29] Meng, Z. & Lu, B. Dust events as a risk factor for daily hospitalization for respiratory and cardiovascular diseases in Minqin, China. *Atmos. Environ.* **41**, 7048–7058 (2007).

[30] Rami, A., Hamidi, M. & Neya, B. N. Atmospheric analysis of dust storms in Sistan region. *J. Atmos. Solar Terr. Phys.* **227**, 105800 (2022).

[31] Hamidi, M. & Roshani, A. Investigation of climate change effects on Iraq dust activity using LSTM. *Atmos. Pollut. Res.* **14**(10), 101874 (2023).

[32] Cheng, Y., Ho, K. F., Lee, S. C. & Law, S. W. Seasonal and diurnal variations of PM1.0, PM2.5, and PM10 in the roadside environment of Hong Kong, China. *Particuology* **4**(6), 312–315 (2006).

[33] Hoffmann, C., Funk, R., Wieland, R., Li, Y. & Sommer, M. Effects of grazing and topography on dust flux and deposition in the Xilingele grassland, Inner Mongolia. *J. Arid Environ.* **72**, 792–807 (2008).

[34] Middleton, N. J. Dust storms in the Middle East. *J. Arid Environ.* **10**, 83–96 (1986).

[35] Draxler, R. R., Gillette, D. A., Kirkpatrick, J. S. & Heller, J. Estimating PM10 air concentrations from dust storms in Iraq, Kuwait, and Saudi Arabia. *Atmos. Environ.* **35**(25), 4315–4330 (2001).

[36] Shao, Y. *Physics and Modelling of Wind Erosion* (Springer, 2008).

[37] Goudie, A. S. & Middleton, N. J. *Desert dust in the Global System* (Springer, 2006).

[38] Hamidi, M., Kavianpour, M. R. & Shao, Y. Numerical simulation of dust events in the Middle East. *Aeol. Res.* **13**, 59–70 (2014).

[39] Hamidi, M., Kavianpour, M. R. & Shao, Y. A quantitative evaluation of the 3–8 July 2009 Shamal dust storm. *Aeol. Res.* **24**, 133–143 (2017).

[40] Li, J., Garshick, E., Al-Hemoud, A., Huang, S. & Koutrakis, P. Impacts of meteorology and vegetation on surface dust concentrations in Middle Eastern countries. *Sci. Total Environ.* **712**, 136597 (2020). 32050389 10.1016/j.scitotenv.2020.136597 PMC7085415

[41] United States Environmental Protection Agency (EPA). National Ambient Air Quality Standards (NAAQS) for Particulate Matter (2021)*.*

